# Factors Influencing the Decision to Undergo Rhinoplasty in Saudi Arabia: Results of a Cross-Sectional Survey

**DOI:** 10.7759/cureus.77817

**Published:** 2025-01-22

**Authors:** Yosra Buhiliga, Hussam Alkhars, Najla Alkilani, Hassan F Alkhars, Abdullah AlAlwan, Ammar J Alsalem, Maha AlQahtani

**Affiliations:** 1 Plastic and Reconstructive Surgery, Dammam Medical Complex, Dammam, SAU; 2 Medicine and Surgery, King Faisal University, Al Hofuf, SAU; 3 Surgery, College of Medicine, King Saud University, Riyadh, SAU; 4 Otolaryngology, King Fahad General Hospital, Al Hofuf, SAU; 5 Plastic and Reconstructive Surgery, King Faisal University, Al Ahsa, SAU; 6 Medicine, King Faisal University, Al Ahsa, SAU

**Keywords:** factors influencing, frequency, rhinoplasty, saudi arabia, self-confidence, social media influence

## Abstract

Background and objective

Rhinoplasty is a popular cosmetic procedure and it is influenced by various social, psychological, and medical factors. Understanding these motivations can aid healthcare providers in offering comprehensive care. In this study, we aimed to investigate the factors influencing the decision to undergo rhinoplasty, as well as previous experience and the factors that led to the decision to undergo the procedure in Saudi Arabia.

Methods

A cross-sectional study was conducted by using an online questionnaire distributed through healthcare networks and social media platforms in Saudi Arabia The study included 717 adult participants. Data on sociodemographic characteristics, lifestyle factors, and motivations to undergo rhinoplasty were collected and analyzed using IBM SPSS Statistics for Windows, Version 27.0 (IBM Corp., Armonk, NY). Descriptive and inferential statistics were used to identify significant associations.

Results

The largest proportion of participants were from the Central Region (235, 32.8%); most were aged 26-35 years (183, 25.5%) and female (401, 55.9%). Social media, especially influencers, played a significant role in the decision to undergo rhinoplasty, with 193 (26.9%) agreeing that social media celebrities influenced them. Bullying (195, 27.2%), aesthetic standards (158, 22.0%), and self-confidence (196, 27.3%) were also key factors that played a role.

Conclusions

This study highlights that several factors, including social media, societal standards, and personal reasons like self-confidence and bullying, influence the decision to undergo rhinoplasty in Saudi Arabia. Regional and socioeconomic disparities further shape these decisions, with younger individuals and high-income groups showing a greater desire and propensity to undergo the procedure.

## Introduction

Rhinoplasty or nasal reshaping surgery is the most common cosmetic procedure performed worldwide. The procedure is also done for medical reasons, such as trauma to the nose and nasal obstruction diseases. It is a highly complicated procedure that requires the service of a highly competent surgeon to achieve optimal results [1]. The most important phase in terms of rhinoplasty is the preoperative stage, when the surgeon would study the patient nose keeping in mind its shape, and surrounding structures, and would decide on what fits best for the patient, to avoid any possible deformity and dissatisfaction [2]. The American Society of Aesthetic Plastic Surgery showed that rhinoplasty accounted for 352,555 procedures among more than 17.7 million total cosmetic surgeries [3]. Rhinoplasty made up 60% of all cosmetic procedures performed In Saudi Arabia in 2019 [4].

Factors that would increase the likelihood of a person undergoing cosmetic procedures were previously reported to be increasing media exposure and poor self-esteem [5]. A previous study conducted in Saudi Arabia found that 65.7% of all individuals going to cosmetic clinics were motivated to undergo a cosmetic procedure due to before-and-after photographs that they have come across related to surgery results [6]. Overall, the decision to undergo rhinoplasty is multifaceted, necessitating a comprehensive understanding of the psychological, social, and cultural influences involved. This study aims to explore the factors influencing the decision to undergo rhinoplasty, as well as previous experience and the factors that led to the decision to undergo the procedure in Saudi Arabia.

## Materials and methods

Study design

We conducted a cross-sectional study from February to December 2024, involving Saudi individuals from various regions. 

Inclusion and exclusion criteria

We included all the participants who were aged 18 years and above of both genders living in Saudi Arabia. We excluded all the participants who were from outside of Saudi Arabia and those aged below 18 years.

Data collection

We initially prepared the study questionnaire in the English language for a better grasp of the medical terms and overall clarity. Then by consulting an Arabic language expert, we translated the questionnaire into Arabic to ensure good participation and understanding among the Saudi population as Arabic is their native tongue. As per Lawshe's method for the validation of the study questionnaire we adhered to, we asked nine consultants and experts in the field of the study to review and comment on the questionnaire. We calculated the content validity ratio, any item with a value below 0.99 was removed. Then, we conducted a pilot study involving 109 participants to evaluate the reliability of the questionnaire. No data from the pilot study were included in the final analysis. 

Via a non-probability convenience sampling, we invited participants who met the inclusion criteria to take part in the study. We published the Arabic version of the study questionnaire as a Google Form Survey on different social media platforms and healthcare networks. The study questionnaire had three main parts: (1) demographical data of the participants; (2) general views on rhinoplasty and the factors influencing the decision to undergo the procedure and the participants' opinions about them; (3) previous rhinoplasty experience and the influencing factor associated with it. Responses were compiled in Microsoft Excel (Microsoft Corp., Redmond, WA) for initial assessment.

Statistical analysis

The statistical analysis for this study was conducted using IBM SPSS Statistics for Windows, Version 27.0 (IBM Corp., Armonk, NY). Descriptive statistics, such as frequencies and percentages, were used to summarize the sociodemographic characteristics and responses to factors influencing the decision to undergo rhinoplasty. Inferential statistics were used to test for significant associations between the factors and the history of undergoing rhinoplasty. Pearson's chi-square test (χ²) was used to assess the relationships between categorical variables, such as region, gender, educational level, work status, monthly income, and marital status. The exact probability test was applied where appropriate to ensure accurate p-value calculations for small sample sizes. Significance levels were set at p<0.05 for all tests. Also, the association between factors influencing the decision and participants' age and gender was tested.

Confidentiality and ethics

We maintained the privacy of the participants and the confidentiality of the data through every step of this study. Ethical approval was obtained from the Research Ethical Committee of King Faisal University (reference code: KFU-REC-2024-DEC-ETHICS2838).

## Results

Table 1 presents the sociodemographic characteristics of the study participants. The highest proportion of participants hailed from the Central Region (235, 32.8%), followed by the Western Region (159, 22.2%), the Northern Region (140, 19.5%), the Eastern Region (109, 15.2%), and the Southern Region (74, 10.3%). Age-wise, the largest group was 26-35 years (183, 25.5%), followed by 36-45 years (173, 24.1%), 46-55 years (153, 21.3%), 18-25 years (113, 15.8%), and above 55 years (95, 13.2%). Regarding gender, the sample consisted of 316 males (44.1%) and 401 females (55.9%). Educational levels varied among participants, with 59 (8.2%) being illiterate, 75 (10.5%) having basic education, 105 (14.6%) holding a diploma, 220 (30.7%) having a university education, and 258 (36.0%) possessing post-graduate qualifications. In terms of work status, 194 (27.1%) were not working, 171 (23.8%) were students, and 352 (49.1%) were employed. As for monthly income, 117 (16.3%) participants reported earning less than 5000 Saudi Riyals (SR), 220 (30.7%) reported between 5000 and 10000 SR, 234 (32.6%) reported between 10000 and 20000 SR, and 146 (20.4%) earned more than 20000 SR. Marital status data showed that 145 (20.2%) participants were divorced or widowed, 285 (39.7%) were single, and 287 (40.0%) were married.

**Table 1:** Sociodemographic characteristics of the study participants (n=717) SR: Saudi Riyal

Demographic data	N	%
Region		
Central Region	235	32.8%
Northern Region	140	19.5%
Eastern Region	109	15.2%
Western Region	159	22.2%
Southern Region	74	10.3%
Age group, years		
18-25	113	15.8%
26-35	183	25.5%
36-45	173	24.1%
46-55	153	21.3%
>55	95	13.2%
Gender		
Male	316	44.1%
Female	401	55.9%
Educational level		
Illiterate	59	8.2%
Basic education	75	10.5%
Diploma	105	14.6%
University education	220	30.7%
Post-graduate	258	36.0%
Work status		
Not working	194	27.1%
Student	171	23.8%
Employed	352	49.1%
Monthly income, SR		
<5000	117	16.3%
5000-10000	220	30.7%
10000-20000	234	32.6%
>20000	146	20.4%
Marital status		
Single	285	39.7%
Married	287	40.0%
Divorced/widowed	145	20.2%

Table 2 presents the factors influencing the decision to undergo rhinoplasty in Saudi Arabia. When asked about their general thoughts on rhinoplasty, 78 participants (10.9%) strongly disagreed, 164 (22.9%) disagreed, 211 (29.4%) were neutral, 184 (25.7%) agreed, and 80 (11.2%) strongly agreed. Regarding the influence of bullying from family and close ones, 82 participants (11.4%) strongly disagreed, 183 (25.5%) disagreed, 167 (23.3%) were neutral, 195 (27.2%) agreed, and 90 (12.6%) strongly agreed. When considering the impact of social media celebrities, 93 participants (13.0%) strongly disagreed, 155 (21.6%) disagreed, 161 (22.5%) were neutral, 193 (26.9%) agreed, and 115 (16.0%) strongly agreed. Regarding the influence of consulting plastic surgeons and obtaining their approval, 85 participants (11.9%) strongly disagreed, 169 (23.6%) disagreed, 170 (23.7%) were neutral, 182 (25.4%) agreed, and 111 (15.5%) strongly agreed. On the subject of aesthetic standards in society driving the decision for rhinoplasty, 94 participants (13.1%) strongly disagreed, 174 (24.3%) disagreed, 170 (23.7%) were neutral, 158 (22.0%) agreed, and 121 (16.9%) strongly agreed. Lastly, regarding rhinoplasty as a way to increase self-confidence, 91 participants (12.7%) strongly disagreed, 181 (25.2%) disagreed, 163 (22.7%) were neutral, 196 (27.3%) agreed, and 86 (12.0%) strongly agreed.

**Table 2:** Factors influencing the decision to undergo rhinoplasty (n=717)

Factors	Strongly disagree		Disagree		Neutral		Agree		Strongly agree	
	N	%	N	%	N	%	N	%	N	%
What do you think about rhinoplasty?	78	10.9%	164	22.9%	211	29.4%	184	25.7%	80	11.2%
A person performs rhinoplasty because of bullying from his family and those close to him	82	11.4%	183	25.5%	167	23.3%	195	27.2%	90	12.6%
A person gets rhinoplasty because social media celebrities influence him	93	13.0%	155	21.6%	161	22.5%	193	26.9%	115	16.0%
People's desire to undergo rhinoplasty increases when they consult plastic surgeons and obtain approval from them	85	11.9%	169	23.6%	170	23.7%	182	25.4%	111	15.5%
A person performs rhinoplasty because of an aesthetic standard in society	94	13.1%	174	24.3%	170	23.7%	158	22.0%	121	16.9%
A person performs rhinoplasty to increase self-confidence	91	12.7%	181	25.2%	163	22.7%	196	27.3%	86	12.0%

**Figure 1:**
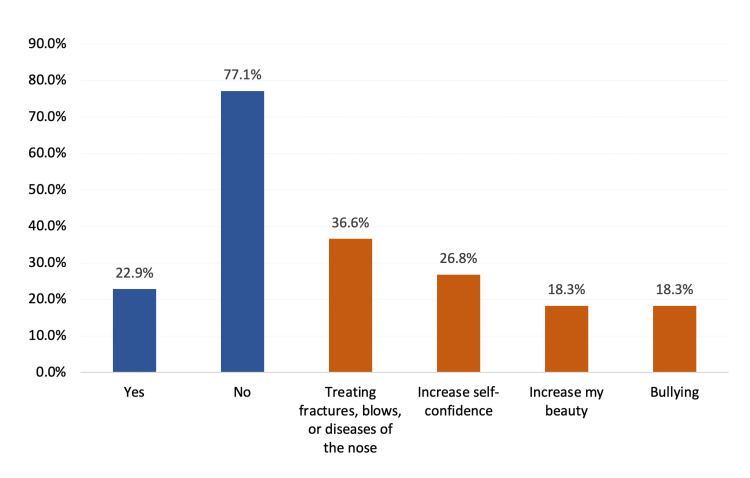
Frequency of and motives behind undergoing rhinoplasty among the study participants

Figure 1 presents the frequency and motives related to rhinoplasty. A significant portion of respondents (77.1%; 553) had never undergone rhinoplasty, while 164 (22.9%) had. Among those who had undergone the procedure, the most common reasons for seeking rhinoplasty included medical needs such as treating fractures, blows, or diseases of the nose (36.6%). Many individuals also cited cosmetic or emotional factors, such as increasing their self-confidence (26.8%) and seeking to enhance their beauty (18.3%). Additionally, bullying was a factor for another 18.3% of respondents.

Table 3 presents the distribution of factors influencing the decision to undergo rhinoplasty by participants' age. Regarding the influence of bullying from family and close ones, 57 (50.4%) of participants aged 18-25 years agreed that it was a factor, while 77 (42.1%) and 74 (42.8%) of participants aged 26-35 years and 36-45 years, respectively, disagreed. In the older age groups (46-55 years and >55 years), 58 (37.9%) and 31 (32.6%), respectively, also disagreed (p=0.014). The influence of social media celebrities on the decision to undergo rhinoplasty showed a significant association with participant age. Among participants aged 18-25, 54.0% agreed that social media celebrities influenced their decision, while this agreement was lower in the 26-35-year (73, 39.9%) and 36-45-year (66, 38.2%) age groups. In contrast, a higher percentage of participants aged 46-55 years (68, 44.4%) and those >55 years (40, 42.1%) also agreed with this statement (p=0.004). Aesthetic standards in society were another significant factor. Among participants aged 18-25 years, 53 (46.9%) agreed that societal aesthetic standards influenced their decision to undergo rhinoplasty, compared to 58 (31.7%) aged 26-35 years and 67 (38.7%) aged 36-45 years. In the older age groups (46-55 years and >55 years), 66 (43.1%) and 35 (36.8%), respectively, agreed (p=0.047). Lastly, increasing self-confidence was identified as a significant factor. Participants aged 18-25 years (53, 46.9%), 26-35 years, (68, 37.2%), 36-45 years, (77, 44.5%), and those >55 years (38, 40.0%) predominantly agreed that undergoing rhinoplasty was driven by a desire to increase self-confidence. However, a higher proportion of participants aged 46-55 years (60, 39.2%) disagreed with this statement (p=0.031).

**Table 3:** Distribution of factors influencing the decision to undergo rhinoplasty by participants' age ^*^P<0.05 (significant). ^^^Exact probability test P: Pearson X^2^ test

Factors		Age group, years										P-value
		18-25		26-35		36-45		46-55		>55		
		N	%	N	%	N	%	N	%	N	%	
What do you think about rhinoplasty?	Disagree	37	32.70%	61	33.30%	62	35.80%	42	27.50%	40	42.10%	0.181
	Neutral	40	35.40%	54	29.50%	49	28.30%	41	26.80%	27	28.40%	
	Agree	36	31.90%	68	37.20%	62	35.80%	70	45.80%	28	29.50%	
A person performs rhinoplasty because of bullying from his family and those close to him	Disagree	25	22.10%	77	42.10%	74	42.80%	58	37.90%	31	32.60%	0.014^*^^
	Neutral	31	27.40%	39	21.30%	32	18.50%	42	27.50%	23	24.20%	
	Agree	57	50.40%	67	36.60%	67	38.70%	53	34.60%	41	43.20%	
A person gets rhinoplasty because social media celebrities influence him	Disagree	32	28.30%	74	40.40%	59	34.10%	41	26.80%	42	44.20%	0.004^*^
	Neutral	20	17.70%	36	19.70%	48	27.70%	44	28.80%	13	13.70%	
	Agree	61	54.00%	73	39.90%	66	38.20%	68	44.40%	40	42.10%	
People's desire to undergo rhinoplasty increases when they consult plastic surgeons and obtain approval from them	Disagree	29	25.70%	67	36.60%	63	36.40%	59	38.60%	36	37.90%	0.478
	Neutral	32	28.30%	46	25.10%	35	20.20%	36	23.50%	21	22.10%	
	Agree	52	46.00%	70	38.30%	75	43.40%	58	37.90%	38	40.00%	
A person performs rhinoplasty because of an aesthetic standard in society	Disagree	33	29.20%	88	48.10%	60	34.70%	53	34.60%	34	35.80%	0.047^*^^
	Neutral	27	23.90%	37	20.20%	46	26.60%	34	22.20%	26	27.40%	
	Agree	53	46.90%	58	31.70%	67	38.70%	66	43.10%	35	36.80%	
A person performs rhinoplasty to increase self-confidence	Disagree	33	29.20%	78	42.60%	66	38.20%	60	39.20%	35	36.80%	0.031^*^
	Neutral	27	23.90%	37	20.20%	30	17.30%	47	30.70%	22	23.20%	
	Agree	53	46.90%	68	37.20%	77	44.50%	46	30.10%	38	40.00%	

Table 4 presents factors influencing the decision to undergo rhinoplasty by participants' gender. For the general opinion about rhinoplasty, 123 (38.9%) males and 119 (29.7%) females disagreed, while 77 (24.4%) males and 134 (33.4%) females were neutral. Agreement was relatively similar between genders: 116 (36.7%) males and 148 (36.9%) females (p=0.009). Regarding the influence of bullying from family and close ones, 129 (40.8%) males and 136 (33.9%) females disagreed, while 72 (22.8%) males and 95 (23.7%) females were neutral. A higher percentage of females (170, 42.4%) agreed compared to males (115, 36.4%), though this difference was not statistically significant (p=0.139). The influence of social media celebrities showed a significant difference between genders. Among males, 122 (38.6%) disagreed, 74 (23.4%) were neutral, and 120 (38.0%) agreed. In contrast, 126 (31.4%) of females disagreed, 87 (21.7%) were neutral, and a higher percentage (188, 46.9%) agreed (p=0.046). Consulting plastic surgeons and obtaining their approval also demonstrated a significant gender difference. Among males, 127 (40.2%) disagreed, 73 (23.1%) were neutral, and 116 (36.7%) agreed. For females, 127 (31.7%) disagreed, 97 (24.2%) were neutral, and a higher percentage (177, 44.1%) agreed (p=0.047). As for the influence of aesthetic standards in society, 130 (41.1%) males and 138 (34.4%) females disagreed, while 72 (22.8%) males and 98 (24.4%) females were neutral. Agreement was reported by 114 (36.1%) males and 165 (41.1%) females, but this difference was not statistically significant (p=0.173). Lastly, the factor of increasing self-confidence was not significantly different between genders. Among males, 127 (40.2%) disagreed, 79 (25.0%) were neutral, and 110 (34.8%) agreed. For females, 145 (36.2%) disagreed, 84 (20.9%) were neutral, and a higher percentage (172, 42.9%) agreed (p=0.083).

**Table 4:** Distribution of factors influencing the decision to undergo rhinoplasty by participants' gender ^*^P<0.05 (significant).^ ^^Exact probability test P: Pearson X^2^ test

Factors	Gender				P-value
	Male		Female		
	N	%	N	%	
What do you think about rhinoplasty?					0.009^*^
Disagree	123	38.9%	119	29.7%	
Neutral	77	24.4%	134	33.4%	
Agree	116	36.7%	148	36.9%	
A person performs rhinoplasty because of bullying from his family and those close to him					0.139
Disagree	129	40.8%	136	33.9%	
Neutral	72	22.8%	95	23.7%	
Agree	115	36.4%	170	42.4%	
A person gets rhinoplasty because he is influenced by social media celebrities					0.046^*^
Disagree	122	38.6%	126	31.4%	
Neutral	74	23.4%	87	21.7%	
Agree	120	38.0%	188	46.9%	
People's desire to undergo rhinoplasty increases when they consult plastic surgeons and obtain approval from them					0.047^*^
Disagree	127	40.2%	127	31.7%	
Neutral	73	23.1%	97	24.2%	
Agree	116	36.7%	177	44.1%	
A person performs rhinoplasty because of an aesthetic standard in society					0.173
Disagree	130	41.1%	138	34.4%	
Neutral	72	22.8%	98	24.4%	
Agree	114	36.1%	165	41.1%	
A person performs rhinoplasty to increase self-confidence					0.083^^^
Disagree	127	40.2%	145	36.2%	
Neutral	79	25.0%	84	20.9%	
Agree	110	34.8%	172	42.9%	

Table 5 presents the factors associated with the history of undergoing rhinoplasty among the study participants. There was a significant association between the history of undergoing rhinoplasty and the participants' region of residence. Participants from the Central Region had the highest proportion of rhinoplasty procedures, with 67 (28.5%) having undergone the surgery compared to 168 (71.5%) who had not; the Northern Region had the lowest proportion, with 19 (13.6%) having undergone rhinoplasty and 121 (86.4%) who had not (p=0.005). Monthly income also showed a significant association with the decision to undergo rhinoplasty. Participants with a monthly income of more than 20000 SR had the highest proportion of rhinoplasty procedures, with 49 (33.6%) having undergone the surgery compared to 97 (66.4%) who had not. This proportion decreased with lower income levels, with only 13 (11.1%) of participants earning less than 5000 SR having undergone rhinoplasty compared to 104 (88.9%) who had not (p=0.0003).

**Table 5:** Factors associated with history of undergoing rhinoplasty among the study participants ^*^P<0.05 (significant). ^^^Exact probability test P: Pearson X^2^ test

Factors	Have you ever had rhinoplasty?				P-value
	Yes		No		
	N	%	N	%	
Region					0.005^*^
Central Region	67	28.5%	168	71.5%	
Northern Region	19	13.6%	121	86.4%	
Eastern Region	29	26.6%	80	73.4%	
Western Region	38	23.9%	121	76.1%	
Southern Region	11	14.9%	63	85.1%	
Age group, years					0.0002^*^
18-25	33	29.2%	80	70.8%	
26-35	57	31.1%	126	68.9%	
36-45	40	23.1%	133	76.9%	
46-55	22	14.4%	131	85.6%	
>55	12	12.6%	83	87.4%	
Gender					0.027^*^
Male	60	19.0%	256	81.0%	
Female	104	25.9%	297	74.1%	
Educational level					0.174^^^
Illiterate	14	23.7%	45	76.3%	
Basic education	15	20.0%	60	80.0%	
Diploma	21	20.0%	84	80.0%	
University education	42	19.1%	178	80.9%	
Post-graduate	72	27.9%	186	72.1%	
Work status					0.0003^*^
Not working	28	14.4%	166	85.6%	
Student	34	19.9%	137	80.1%	
Employee	102	29.0%	250	71.0%	
Monthly income					0.0003^*^
<5000 SR	13	11.1%	104	88.9%	
5000-10000 SR	49	22.3%	171	77.7%	
10000-20000 SR	53	22.6%	181	77.4%	
>20000 SR	49	33.6%	97	66.4%	
Marital status					0.072
Single	55	19.3%	230	80.7%	
Married	78	27.2%	209	72.8%	
Divorced/widowed	31	21.4%	114	78.6%	

## Discussion

The study highlighted several factors that influence the decision to undergo rhinoplasty among participants in Saudi Arabia. Rhinoplasty, or nose reshaping surgery, is a popular cosmetic procedure driven by various social and psychological factors. A significant number of participants either had neutral or positive views on rhinoplasty. This shows a relatively open attitude toward cosmetic surgery within the study population, which aligns with global trends where cosmetic surgery is becoming more accepted [7-9]. Bullying from family and close ones emerged as a notable influence, with a substantial portion of participants agreeing that this factor played a role. The impact of bullying on the decision to undergo cosmetic surgery is well-documented. For instance, previous studies revealed that peers significantly influence body image, and nearly half of adults seeking cosmetic surgery report having experienced teasing or bullying about their appearance during childhood or adolescence [10-12].

Social media celebrities were another significant influence. Many participants agreed that the impact of social media played a role in their decision-making process. This finding is consistent with previous research indicating that exposure to idealized images on social media can lead to a higher interest in cosmetic surgery [13]. A third study by Arab et al. revealed that about half of respondents were influenced by social media to consider cosmetic procedures, with 51.4% following plastic surgeons online. Most respondents (53.2%) reported using social media for over five hours a day. The findings were statistically significant [14]. In Saudi Arabia, Taha et al. revealed that 28.4% of respondents were influenced by social media, particularly by celebrities or trusted figures, in deciding to undergo rhinoplasty. Snapchat was the most influential platform (43.4%), followed by Twitter (22.97%) and Instagram (12.09%). Social media's impact was notably higher in the southern region of Saudi Arabia (29.3%) compared to the western region (27.8%) [15]. Also, according to a study conducted previously in the central region of Saudi Arabia among patients who underwent rhinoplasty, most of the study sample were influenced by celebrity pictures before and after [16]. 

Consulting with plastic surgeons and obtaining their approval also influenced many participants. Plastic surgeons play a crucial role in shaping patients' decisions by providing expert opinions and recommendations. This is supported by other studies that highlight the importance of medical professionals in the decision-making process for cosmetic surgery [17,18]. Also, societal beauty standards were identified as a significant driver for rhinoplasty. The desire to conform to these standards can pressure individuals into seeking cosmetic enhancements. Research in the United States has shown that societal expectations and cultural beauty standards are strong motivators for undergoing rhinoplasty [19]. Finally, the desire to increase self-confidence was a significant factor. Cosmetic surgery is often pursued to enhance self-esteem and body image. Studies have shown that individuals undergoing rhinoplasty often report increased self-confidence and satisfaction with their appearance post-surgery [20]. A study by Hashemi et al. also found that improving self-esteem was a primary motivation for patients seeking rhinoplasty [21]. In Saudi Arabia, Obeid et al. reported a similar improvement in self-esteem after rhinoplasty [22]. Another study revealed that self-esteem was among the motivators to undergo rhinoplasty in Saudi Arabia [23].

Regarding frequency and reasons to undergo rhinoplasty, a significant majority of the study participants had never undergone rhinoplasty, while those who had cited medical needs, such as treating nasal fractures or diseases, as the most common reason. Cosmetic reasons, including the desire to enhance self-confidence and beauty, were also significant motivators. Bullying was another notable factor, aligning with global findings where social pressure and bullying contribute to the decision to seek cosmetic surgery. A study by Alsubeeh et al. revealed that 44.7% of individuals were considering rhinoplasty revision. The primary motivation was the desire for further aesthetic improvement in an already acceptable result (50.16%) [24].

This study stands out as it delves into factors influencing the decision to undergo rhinoplasty in Saudi Arabia, as well as the fact that it included participants from all the different regions of the country. However, we have faced certain limitations when conducting the study. Firstly, the fact that we used an online questionnaire via Google Form survey and distributed it through social media platforms and shopping malls, may have led to several people not being able to participate in the survey. Secondly, the sample size for each region varied. Hence, the findings may not be fully representative of the entire Kingdom's population.

## Conclusions

The decision to undergo rhinoplasty among participants in Saudi Arabia is influenced by a combination of social, psychological, and medical factors. The findings are consistent with global trends, where bullying, social media influence, consultations with surgeons, societal beauty standards, and the desire for increased self-confidence play pivotal roles in the decision-making process. One-fifth of the participants underwent rhinoplasty mainly to treat nasal trauma or disease and to improve self-confidence. It is recommended that healthcare providers adopt a comprehensive approach when consulting with patients considering rhinoplasty. This includes providing thorough information about the expected outcomes, potential risks, and complications, as well as using tools such as computer imaging to help patients visualize the results. Building strong rapport and understanding patients' specific concerns can help address their motivations and expectations effectively.
